# Coupling Coordination and Influencing Factors among Tourism Carbon Emission, Tourism Economic and Tourism Innovation

**DOI:** 10.3390/ijerph18041601

**Published:** 2021-02-08

**Authors:** Yue Pan, Gangmin Weng, Conghui Li, Jianpu Li

**Affiliations:** School of Economics and Management, Yanshan University, Qinhuangdao 066004, China; panyue_e@163.com (Y.P.); liconghui1996@126.com (C.L.); lijianpuysu@163.com (J.L.)

**Keywords:** tourism carbon emission efficiency, tourism economy, tourism innovation, ternary coupling coordination model, spatiotemporal evolution, driving factors

## Abstract

To discuss the coupling coordination relationship among tourism carbon emissions, economic development and regional innovation it is not only necessary to realize the green development of tourism economy, but also great significance for the tourism industry to take a low-carbon path. Taking the 30 provinces of China for example, this paper calculated the tourism carbon emission efficiency based on the super-efficiency Slacks based measure and Data envelope analyse (SBM-DEA) model from 2007 to 2017, and on this basis, defined a compound system that consists of tourism carbon emissions, tourism economic development and tourism regional innovation. Further, the coupling coordination degree model and dynamic degree model were used to explore its spatiotemporal evolution characteristics of balanced development, and this paper distinguished the core influencing factors by Geodetector model. The results showed that (1) during the study period, the tourism carbon emission efficiency showed a reciprocating trend of first rising and then falling, mainly due to the change of pure technical efficiency. (2) The coupling coordination degree developed towards a good trend, while there were significant differences among provinces, showing a gradient distribution pattern of decreasing from east to west. Additionally, (3) the core driving factors varied over time, however, in general, the influence from high to low were as follows: technological innovation, economic development, urbanization, environmental pollution control, and industrial structure. Finally, some policy recommendations were put forward to further promote the coupling coordination degree.

## 1. Introduction

With the increasingly severe global warming problem, energy conservation, emission reduction, and low-carbon has become the general consensus of sustainable economic development [1]. The key is to build an ideal model of high-quality economic development by reducing energy consumption, carbon emissions and ecological environment pressure [2]. Studies have shown that tourism carbon emissions account for about 8% of total carbon emissions and are gradually increasing at an average rate of 3.2%. If no measures are taken, the global tourism carbon emissions will increase by 130% by 2035 [3,4,5]. As one of the countries under the United Nations Framework Convention on Climate Change, the Chinese government promised to reduce carbon emissions per unit of GDP by 60% to 65% by 2030 compared with 2005. The tourism economy, as a strategic pillar industry for China’s economic development, achieved total tourism revenue of 6.63 trillion CNY in 2019 and it grew by 11% over the last year. The rapid growth of the tourism economy meant that carbon emissions have also increased correspondingly. Therefore, how to realize the low-carbon development of tourism while ensuring the sustainable growth of tourism economy is an important subject to be discussed urgently.

The coordinated development of economic development and regional innovation is the foundation and motivation for the healthy and sustainable development of regional tourism. As an important part of economic development and regional innovation, the reduction of tourism carbon emissions is the key to improving the carbon emission efficiency of tourism. The 19th National Congress of the Communist Party of China (CPC) pointed out that China should focus on solving prominent environmental problems, establish and improve the economic system of green, low-carbon, and circular development, which provided policy guidance for green development of China’s tourism economy. However, at present, the contradiction between carbon emission, economic development and regional innovation of tourism industry in various provinces is still prominent, and the problem of imbalanced development between provinces is severe. Therefore, it is of great significance to study the coupling coordination of carbon emission, economic development and regional innovation of tourism industry in various provinces for China’s tourism energy conservation and emission reductions, and regional green coordinated development.

The tourism carbon emission–economic development–regional innovation system is an organic whole with complex content structure and interactive coupling relationship formed by three major systems of tourism carbon emission, tourism economic development and tourism regional innovation. A thorough understanding of the coupling mechanism among three major systems is the primary prerequisite to better solve the problem of coordinated development of tourism carbon emission–economic development–regional innovation. Firstly, there is an interaction between tourism carbon emissions and economic development. The development of tourism economy provides financial support for carbon emission reduction and alleviates the increasingly severe environmental pressure by improving carbon emission efficiency and promoting energy conservation and emission reduction. The increase in tourism carbon emissions means that the higher the GDP per unit of carbon emissions output, the more beneficial to economic development. On the contrary, economic development consumes more energy and restricts economic development [6]. Secondly, tourism carbon emissions and regional innovation are mutually influenced. The impact of regional innovation on tourism carbon emissions is mainly achieved through technological innovation. Through the effective allocation of resources, the level of energy utilization is improved, and the development of clean and renewable energy sources can reduce carbon emissions. Energy consumption and carbon emissions are closely related to the technological level. The more carbon emissions produced by per unit energy consumption means the lower the technological level of energy utilization, so the technological innovation of energy conservation and emission reduction should be accelerated. Finally, economic development and regional innovation influence each other. Economic development provides economic support for regional innovation, and the development of tourism economy promotes the accumulation and diffusion of relevant resource elements within the region, providing an excellent soft and hard environment for innovation. Regional innovation provides a fundamental driving force for economic development. By adjusting and optimizing the structure of factors, researching and developing new products and services, promoting the conversion of the new and old kinetic energy of economic development, the quality and efficiency of development will be improved. In other words, economic development is the basic guarantee of regional innovation and carbon emission reduction, regional innovation is the source of power, and tourism carbon emissions is an important link. While, there is still a lack of research on the overall interaction among tourism carbon emissions, regional innovation and economic development, and the existing research is only limited to the interaction and integration between the two, such as tourism carbon emissions and regional innovation [7], tourism carbon emissions and economic development [8,9], or regional innovation and economic development [10].

Therefore, this paper aimed to establish the evaluation index system of tourism carbon emission, economic development and regional innovation, and discussed the interaction among them. For the first time, this paper empirically analyzed the coupling and coordination of three major systems of tourism industry at provincial level. On the basis, the influencing factors were explored, and the research results provided a basis for the formulation of sustainable tourism development policies.

## 2. Literature Review

The research on tourism carbon emissions began in the 1990s. Gössling used the “bottom-up” method for the first time to measure the carbon emissions of tourism transportation, tourism accommodation and other tourism activities [11]. The basic idea was to first obtain the total energy consumption of tourism or tourism consumption, then the carbon emissions of tourism industry were calculated according to the carbon emission coefficients of various energy sources. Subsequently, the research results of tourism carbon emissions based on this method gradually increased. In terms of research scope, it is carried out from the perspectives of the country [12], provinces [13], cities [14], and scenic spots [15]. In terms of research content, it mainly involves three parts: tourism transportation [16], tourism accommodation [17] and tourism activities [18]. In contrast, there are relatively few studies on tourism carbon emissions using the “top-down” approach. Perch-Nielsen used the method to measure the greenhouse gas emission intensity in the Switzerland tourism sector [19]. Based on the balance sheet of energy consumption and referring to the concept of “tourism consumption stripping coefficient”, Xie et al. constructed a method in line with the measurement of tourism carbon emissions in China and made a comparative analysis on the carbon emissions of the tourism industry in Jiangsu, Zhejiang and Shanghai [20]. Based on the “top-down” approach, some scholars combined with tourism satellite account and input–output model to measure tourism carbon emissions and revealed the balance between tourism economic benefit and environmental pollution [21,22].

The analysis of the driving factors of tourism carbon emissions is the key to achieving low-carbon development of the tourism industry [23], which is of great significance to the formulation of emission reduction policies. Scholars have conducted abundant studies on the influencing factors of tourism carbon emissions. It mainly involved the IPAT model [24], Hi_PLS model [25], STIRPAT model [26], SDA [27,28], LMDI decomposition method, etc., and the LMDI decomposition method was the most common. Its basic principle was to decompose the influencing factors into energy structure, energy intensity, consumption level and tourist scale, etc., and on this basis, their contribution degree to the carbon emissions of the tourism industry was calculated [29,30]. In addition, Gössling studied the impact of market composition in 11 different countries on the total amount and intensity of carbon emissions [31]. Taking five national parks in Taiwan as an example, Lin found that different tourist destinations had different carbon emissions from tourism transportation affected by travel distance and traffic patterns. Finally, the scenario analysis method was used to draw the conclusion that increasing traffic load, taking buses, and choosing medium and short-distance tourism destinations could reduce carbon dioxide emissions [32]. Jin used GWR to find the determinants of the carbon emission flow of county self-driving tours in Jiangsu Province, namely, per capita GDP, scores of scenic spots and total resident population showed spatial differences in the province [33].

With the rapid development of tourism, some scholars began to explore the relationship between tourism economy and carbon emissions. It mainly involved decoupling, coupling, Granger causality test, EKC, etc. Tang used a decoupling index to analyze the decoupling effect of China’s tourism economy and carbon dioxide and found that the state of decoupling alternated between negative decoupling and weak decoupling [34]. Katircioglu took Cyprus as an example, used the Granger causality test and found that international tourism was the main catalyst for increasing energy consumption and carbon dioxide emissions [35]. Later scholars combined the decoupling index and Granger causality test to explore the relationship between tourism economy and carbon emissions [36]. Sherafatian-Jahromi found that tourism in Southeast Asia had a long-term impact on carbon dioxide emissions, and the relationship between them showed an inverted “U” shape, and tourism development had an environmental Kuznet curve [37]. Bi combined with the spatial econometric model to discuss the impact of tourism on carbon emissions. It is found that there is a significant inverted “U” shaped relationship between them, and tourism has an obvious spatial lag effect on carbon emissions, which is also an inverted “U” shaped relationship [38]. Paramati used panel econometric techniques to find that tourism played an important role in promoting economic growth, while tourism’s effect on CO_2_ was largely dependent on sustainable tourism policies [39].

To sum up, the current studies of domestic and foreign scholars on tourism carbon emissions are becoming more abundant, but there are still the following shortcomings: there are relatively many discussions on the relationship between tourism carbon emissions and economic development. However, there is a lack of measurement of tourism carbon emissions efficiency indicators. Moreover, the efficiency index which is comprehensively measured by multiple indicators can more easily reflect the true level of regional tourism carbon emission development. Besides, there are many studies on the coupling and decoupling relationship between tourism carbon emissions and economic development, but the indicator of regional innovation is less involved and the research on the three-dimensional coupling coordination of tourism carbon emissions israther little.

Therefore, based on the perspective of system science, this paper measured the carbon emission efficiency of tourism, decomposed it and incorporated it into the tourism carbon emission system; innovatively analyzed the spatial-temporal evolution of the coupling coordination degree of tourism carbon emission, economic development and regional innovation; with the help of geographical detector, we could deeply explore its influencing factors which provided theoretical reference and decision-making basis for China’s tourism carbon emission reduction, economic development and regional innovation.

## 3. Materials and Methods

### 3.1. Index System

#### 3.1.1. The Index Selection of Tourism Carbon Emission Efficiency

Referring to existing studies [40,41], three index variables of capital, labor force and energy were selected as input variables, the total tourism revenue was taken as the expected output, and the tourism carbon emissions were taken as the undesired output.

Among them, the capital element was represented by the stock of fixed capital and its estimation method adopted the perpetual inventory method. Referring to the research of Hall et al. [42], taking 2007 as the base period, the capital stock of each province during the study period was calculated. Although this index is relatively macro, it can better reflect the characteristics of the tourism industry with a strong correlation. The labor factor was represented by the number of employees in the tertiary industry. Although this index is too general, it can fully reflect the comprehensive nature of tourism industry. The energy factor was characterized by the energy consumption of tourism. Since there are no separate statistics on tourism energy consumption in China’s energy statistics, this paper makes use of the regional tourism development coefficient [43]. That is, the total tourism revenue accounts for the proportion of the tertiary industry’s GDP, and the energy consumption of tourism industry is extracted from the various energy terminal consumption of the tertiary industry. The formula is as follows:

(1) $$E_{it}=E_{ij}\times R_{t}$$

where *E_it_* is the energy consumption of tourism industry; *E_ij_* is the terminal consumption of various energy in the tertiary industry; *R_t_* is the tourism development coefficient of each region. On this basis, the carbon emissions of tourism industry can be obtained through further calculations.

(2) $$C_{i}=\sum_{i=1}^{n}E_{it}f_{j}k$$

where *C_i_* represents the carbon emissions of tourism industry in each province; *f_j_* is the standard coal conversion coefficient of type *j* energy (Table 1); *K* is the carbon dioxide emissions of per unit standard coal; referring to the existing research results [44], we set *K* = 2.45.

#### 3.1.2. The Index Selection of Carbon Emission, Economic Development and Regional Innovation System

Based on relevant research results [5,45,46], the construction of the index system followed the principles of scientificity, accessibility and effectiveness. Starting from the efficiency and current situation of the tourism industry’s carbon emissions, this paper selected seven indicators to calculate the comprehensive index of tourism carbon emissions. From the perspective of the economic scale, economic structure and economic vitality, nine indicators were selected to measure the comprehensive index of tourism economic development. Starting from innovation input, innovation output and innovation benefits, nine indicators were selected to calculate the comprehensive index of tourism regional innovation. The main indicators of the three systems are shown in Table 2. Among them, the proportion of tourism R&D expenditure in GDP and the amount of tourism fixed asset investment were obtained by multiplying the total R&D expenditure and the total fixed asset investment of the whole society by the ratio of the total tourism revenue to the total national economic output value, respectively. The granted amount of tourism invention patents was sorted through the Innojoy patent search engine with “tourism” as the keyword. The entropy weight method was used to calculate the weight of each index and obtain the comprehensive evaluation index of each system.

### 3.2. Data Collection

Due to the lack of data in Tibet, Taiwan, Hong Kong and Macao, the data required for this study were panel data from 30 provinces in China, and the research period was from 2007 to 2017. The original data were mainly obtained from the China Energy Statistical Yearbook, China Tourism Statistical Yearbook, China Statistical Yearbook on Science and Technology, China Statistical Yearbook from 2008 to 2018. Besides, some data were obtained by querying the Innojoy patent search engine. When calculating the efficiency of tourism carbon emission, the lag effect of input and output was ignored. The total tourism revenues were composed of domestic tourism revenues and foreign exchange revenues. Additionally, tourism foreign exchange revenue was calculated according to the exchange rate of USD to RMB over the years. For a small number of missing values, the weighted average method was used to complete them.

### 3.3. Methods

#### 3.3.1. Super Efficiency SBM-DEA Model

Data envelopment analysis is a systematic analysis method based on the comparison of relative efficiency between the evaluated objects. This article used the SBM model proposed by Tone that considers undesired output to measure the carbon emission efficiency of the tourism industry in China. The model which has obvious superiority and wide application can not only fully consider the undesired outputs of each decision unit, but also solve the slack problem of input and output variables. Since the model has matured, it will not be repeated here [47].

#### 3.3.2. Coupling Coordination Degree Model

Based on the concept of capacity coupling and the model of capacity coupling coefficient in physics, this paper constructed a coupling model of tourism carbon emission, economic development and regional innovation [48].

(3) $$C={\left\{\frac{f\left(x\right)*g\left(y\right)*h\left(z\right)}{{\left[\frac{f\left(x\right)+g\left(y\right)+h\left(z\right)}{3}\right]}^{3}}\right\}}^{3}$$

where, *C* is the coupling degree and its value is between 0–1 The larger the value of *C*, the smaller the degree of dispersion of the three systems and the higher the coupling degree. *f*(*x*), *g*(*y*), *h*(*z*), respectively, represents the comprehensive evaluation value of tourism carbon emission system, economic development system and regional innovation system.

Since the coupling degree can only describe the size of the interaction between systems, it cannot reflect the level of coordinated development between systems. Therefore, this paper introduced the coupling coordination degree model to analyze and study the coordination degree among systems more deeply and concretely. The calculation formulas are as follow:

(4) $$D=\sqrt{C\times T}$$

(5) $$T=\alpha f\left(x\right)+\beta g\left(y\right)+\gamma h\left(z\right)$$

where, *D* is the coupling coordination degree, *T* is the comprehensive evaluation index of three major systems, *α*, *β*, *γ* are undetermined coefficients and *α* + *β* + *γ* = 1. Considering that tourism carbon emission, economic development and regional innovation are equally important, the paper takes *α* = *β* = *γ* = 1/3. Referring to the existing research results [49], the coordinated development degree of tourism carbon emission–economic development–regional innovation is divided into 10 categories, as shown in Table 3.

#### 3.3.3. Dynamic Degree

In order to measure the size and rate of the change in the coupling coordination degree of carbon emission–economic development–regional innovation system in China’s tourism industry, this paper introduces the dynamic degree to objectively and quantitatively describe the dynamic changes of the coupling coordination development [50]. The specific formula is as follows:

(6) $$K=\frac{D_{t}-D_{0}}{D_{0}}\times \frac{1}{T}\times 100\%$$

where, *K* represents the dynamic degree of coupling coordination development of the three major tourism systems during the study period. *D_t_* and *D*_0_, respectively, represent the coupling coordination degrees of three major tourism systems in the initial and final research period. *T* represents the study period.

#### 3.3.4. Geographical Detector

The geographical detector is a set of statistical methods to detect spatial differentiation and reveal the driving forces behind it. As the geographical detector is less restricted by the premise, they have a strong universality in exploring the formation mechanism and influencing factors of spatial heterogeneity of geographic objects [51]. This paper introduced the factor detection of the geographical detector and analyzed the determining force of influencing factors on the coupling coordination degree of three systems during each study period. The formula model is as follows:

(7) $$P_{D.U}=1-\frac{1}{n\sigma^{2}}\sum_{i=1}^{m}n_{i}\sigma_{i}{}^{2}$$

where, *P_D.U_* represents the influence of the driving factors of coupling coordination degree, *n* is the number of provinces (*n* = 30), *m* is the number of driving factors (*m* = 5), *n* and *n_i_* are the number of sample units in the entire region and the number of sample units in the subregion, respectively, *σ*^2^ and *σ_i_*^2^ are the variances of the change of the coupling coordination degree in the whole region and the variances of the change of the coupling coordination degree in the subregion, respectively.

## 4. Results

### 4.1. Analysis on Carbon Emission Efficiency of the Tourism Industry

#### 4.1.1. An Overall Analysis of the Carbon Emission Efficiency of Tourism Industry

**Figure 1:**
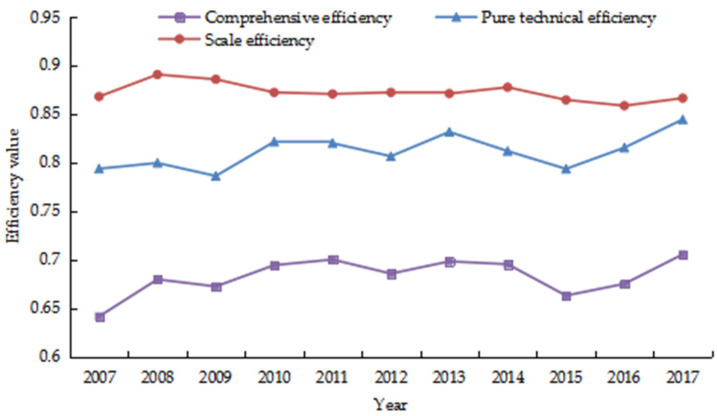
The overall change trend of carbon emission efficiency in China’s tourism industry.

The super-efficiency SBM-DEA model was used to calculate the carbon emission efficiency of China’s tourism industry from 2007 to 2017 (Figure 1). As can be seen from Figure 1, the average value of comprehensive efficiency of tourism carbon emissions in China from 2007 to 2017 was 0.6828. The comprehensive efficiency was further decomposed into pure technical efficiency and scale efficiency, which were 0.8112 and 0.8724, respectively. It can be seen that the current comprehensive efficiency, pure technical efficiency, and scale efficiency of China’s tourism industry have not reached the production frontier and there is still much room for improvement in tourism carbon emission reduction. In terms of the change extent, the comprehensive efficiency of tourism carbon emissions increased from 0.6415 in 2007 to 0.7054 in 2017, with an increase of 9.96%. The pure technical efficiency increased from 0.794 in 2007 to 0.8443 in 2017, with an increase of 6.39%. However, the scale efficiency decreased from 0.8678 in 2007 to 0.8662 in 2017, with a decrease of 0.18%. In terms of the changing trend, all three of them showed an upward–downward reciprocating trend. Among them, the comprehensive efficiency and the pure technical efficiency had a same changing trend during the study period. While except for showing the same trend in 2007–2009 and 2016–2017, the scale efficiency showed an opposite trend in other years. This showed that the changes in carbon emission efficiency of tourism industry in China were mainly caused by the improvement or reduction of technical management level, but had little relationship with the production scale.

#### 4.1.2. Spatial Difference in Carbon Emission Efficiency of Tourism Industry

In order to further reveal the regional differences in carbon emission efficiency level of China’s tourism industry, the average value of tourism carbon emission efficiency in 30 provinces was calculated (Table 4). It can be seen from Table 4 that the tourism carbon emission efficiency in Tianjin, Jiangsu, Guangdong, and Guizhou were greater than 1, which were in the frontier of production. Among them, the level of economic development and the capacity of technological innovation in Tianjin, Jiangsu and Guangdong have provided favorable support for tourism carbon emission efficiency. As a major province of tourism resources, Guizhou had a late start in tourism, poor infrastructure and insufficient optimization of technology application. However, in recent years, with the advancement of the goal of high-quality tourism development, its tourism industry is developing in a spurt with a substantial increase in economic income and significant improvement in carbon emission efficiency. Fujian and Henan were at a relatively efficient level of development. The carbon emission efficiency of tourism in Hunan, Hubei, Jiangxi, Anhui, Shanghai, Sichuan, and Guangxi were at a medium level. In these provinces, the pure technical efficiency was the main determinant of tourism carbon emission efficiency. Therefore, improving the level of technical management and optimizing the allocation and utilization efficiency of various resource elements will be the focus of their future efforts. The comprehensive carbon emission efficiency of tourism in Yunnan, Shaanxi, Xinjiang, and Qinghai were far lower than the national average of 0.6828. With the exception of Hainan, Gansu, Qinghai, Ningxia, and Xinjiang, where the scale efficiency values need to be improved, the scale effects of tourism carbon emissions in other provinces have been fully brought into play. For this reason, these five regions should actively expand the scale of production and increase the input of tourism capital elements to improve their carbon emission efficiency.

Through the above analysis, it is found that carbon emission efficiency is directly related to the level of economic development and regional innovation capacity. Thus, the comprehensive efficiency, pure technical efficiency and scale efficiency of tourism carbon emissions will be incorporated into in the tourism carbon emission system and the coupling coordination analysis will be undertaken with the comprehensive index of economic development and the comprehensive index of regional innovation to explore its spatiotemporal evolution characteristics.

### 4.2. The Spatiotemporal Evolution of the Coupling Coordination Degree of Three Tourism Systems

#### 4.2.1. The Temporal Evolution of Coupling Coordination Degree

**Figure 2:**
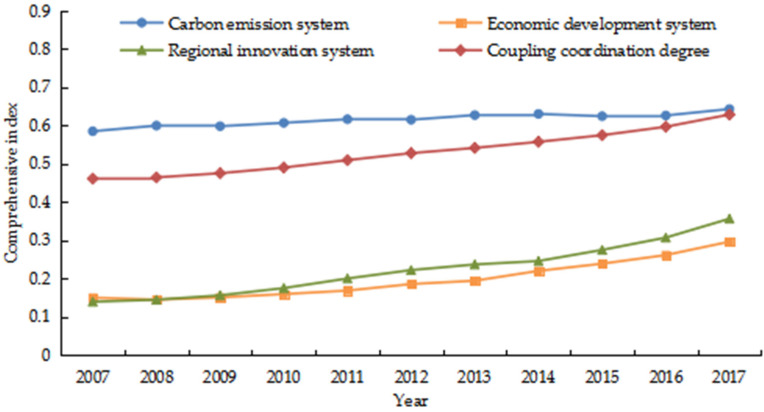
The spatiotemporal evolution of the coupling coordination degree.

According to the coupling coordination degree model, the comprehensive index and coupling coordination degree of carbon emission, economic development and regional innovation of China’s tourism industry from 2007 to 2017 were calculated (Figure 2). (1) From the perspective of the comprehensive index of tourism carbon emission, the development level of carbon emission system in China’s tourism industry presented a reciprocating trend of first rising and then falling. The value fluctuated between 0.5854 and 0.6434, and the stage characteristics were not obvious. This indicated that the carbon emission system of China’s tourism industry was relatively stable and remained at a relatively high level. (2) From the perspective of the comprehensive index of tourism economic development, the development level of this system decreased slightly in 2008, but showed a steady and slow increase in other years. The growth rate has accelerated since 2013 and the value was stable at 0.2969 which showed that the economic system of China’s tourism industry was developing well at this stage, but the level was still poor. (3) From the perspective of the comprehensive index of tourism regional innovation, the development level of regional innovation system showed a continuous upward trend which increased from 0.1399 in 2007 to 0.3569 in 2017, with an increase of 0.2170. It showed that the development trend of regional innovation capability in China’s tourism industry was good, but there was still great room for improvement. (4) From the perspective of the coupling coordination degree, the three systems of China’s tourism industry showed a coupling optimization trend. The type of coupling coordination has changed from approaching imbalance in the early stage to primary coordination in the final stage. It showed that during the study period, China has achieved remarkable results in optimizing the energy consumption structure of tourism industry, strengthening the green development of tourism economy and accelerating technological innovation in energy conservation and emission reduction.

#### 4.2.2. The Spatial Evolution of Coupling Coordination Degree

**Figure 3:**
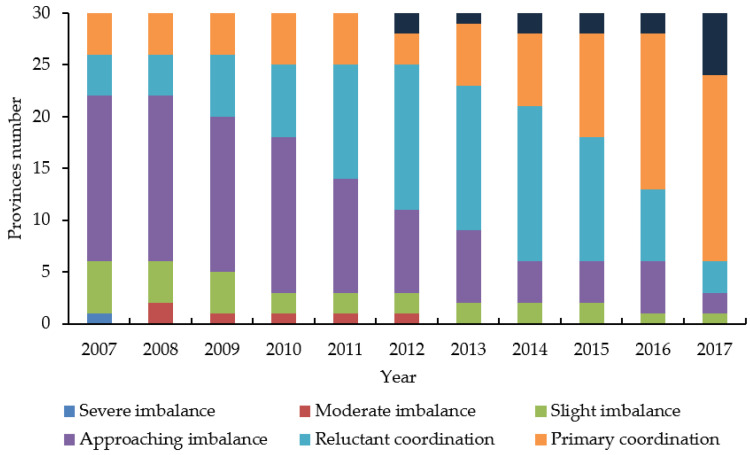
Changes of the coupling coordination rank.

(1) In this paper, the histogram was used to visually display the changes in the number of provinces of each rank type, so as to further understand the rank evolution characteristics of the coupling coordination degree in tourism major systems (Figure 3).

From the perspective of the rank distribution of coupling coordination degree, the coupling coordination degree of China’s tourism carbon emission–economic development–regional innovation crossed five ranks in 2007: severe imbalance, slight imbalance, approaching imbalance, reluctant coordination, and primary coordination. At this time, most provinces in China were still in a state of approaching imbalance and the coupling coordination degree of three systems was poor which needed to be further improved. In 2008, the coupling coordination degree of provinces with severe imbalance developed for the better, the number of provinces of this type dropped to zero and the type of moderate imbalance began to appear. In 2012, the coupling coordination degree of China’s three major systems crossed six ranks of moderate imbalance, slight imbalance, approaching imbalance, reluctant coordination, primary coordination, intermediate coordination. Among them, the provinces in a state of reluctant coordination were the most distributed, accounting for 46.67% of the total. In 2013, the number of provinces with moderate imbalance decreased to zero. Since then, the three major systems of China’s tourism industry crossed five ranks: slight imbalance, approaching imbalance, reluctant coordination, primary coordination, and intermediate coordination. Simultaneously, the main distribution type of provinces in China rose from reluctant coordination to primary coordination. It can be seen that the number of provinces in different ranks has changed during the study period. The major rank type has undergone a transition from approaching imbalance—reluctant coordination—primary coordination which means the coupling coordination degree is developing in a good direction.

**Figure 4:**
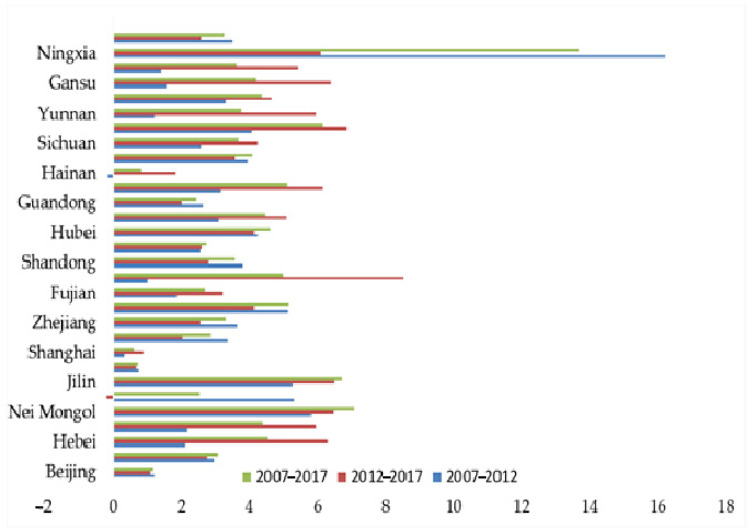
The dynamic degree of coupling coordination development.

The dynamic degree was used to measure the changes in coupling coordination index of each province in China from 2007 to 2012, 2012 to 2017, and 2007 to 2017 (Figure 4). It can be seen from Table 5 that from 2007 to 2017, the dynamic degree of the coupling coordination level in each province was low, that is, the degree of convergence was high. From the perspective of each research stage, from 2007 to 2012, the largest dynamic change was 16.24% in Ningxia and the smallest dynamic change was −0.17% in Hainan. In the direction of change, only Hainan had a negative value, while the rest of the provinces had a positive value. This showed that at this stage, except for the decline in the coupling coordination index of Hainan, the coupling coordination degree of other provinces showed an increasing trend compared to 2007. From 2012 to 2017, the dynamic degree of Liaoning was −0.22%, which is the closest to zero. This indicated that the space for the rise and fall of the coupling coordination degree was to be extremely limited. In terms of the direction of change, only the coupling coordination degree of Liaoning province in 2017 showed a downward trend compared with 2012. In general, from 2007 to 2017, the rate of change among provinces in China was not much different and the change direction of dynamic degree among provinces was consistent. This showed that during the study period, the coupling coordination degree of each province in China was showing an upward trend with small variation differences among provinces and significant convergence characteristics.

**Figure 5:**
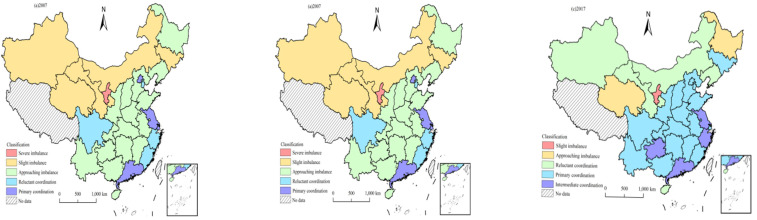
The spatial distribution of the coupling coordination degree of 30 provinces.

In order to reveal the spatial distribution characteristics of the coupling coordination types of tourism three major systems in 30 provinces of China, this paper selected 2007, 2012 and 2017 as the time nodes and used ArcGIS 10.2 software for visual expression (Figure 5).

From the perspective of the spatial distribution pattern of coupling coordination degree, there were obvious regional differences in the coupling coordination degree of three major systems in China’s provincial tourism industry. In 2007, the provinces with severe imbalance and slight imbalance were mostly distributed in the central and western regions, the provinces with approaching imbalance were concentrated in the central region, and the reluctant coordinated and primary coordinated provinces were mainly distributed along the eastern coast. Compared with 2007, some provinces that were originally classified as severe imbalance and moderate imbalance changed to moderate imbalance and approaching imbalance which mainly concentrated in the western region in 2012. The reluctant coordinated ones were mostly distributed in the central and western regions, while the provinces of primary coordination and intermediate coordination were mainly located in the eastern region. In 2017, the coupling coordination degree of most provinces in China was in the state of primary coordination and only a few areas in the west remained in a state of imbalance. During the study period, the areas of coordinated development were mostly eastern provinces such as Jiangsu and Guangdong, which had better economic development. However, the areas of imbalanced recession were in the western region with relatively poor economic development, such as Gansu, Qinghai and Xinjiang. The central region was dominated by two types of imbalance and coordination. It can be seen that there are certain differences in coupling coordination degree of the major three systems in different regions and presented a gradient distribution pattern of “high in the east and low in the west”.

### 4.3. Analysis of Influencing Factors

The level of tourism carbon emission–economic development–regional innovation coupling coordination degree is affected by many factors. On the basis of referring to existing research results [52,53], this paper selected independent variables from five aspects: economic development level, urbanization level, industrial structure, technological innovation level, regional environmental pollution control level. Additionally, they were represented by per capita GDP (X1), the proportion of urban population (X2), the proportion of tertiary industry in GDP (X3), R&D expenditure (X4) and investment in environmental pollution control (X5). Then, the detection result q value of each influencing factor was obtained with the help of geographical detector (Table 5).

Table 5 shows that (1) the impact of the economic development level showed a downward trend. In 2009, the impact on the coupling coordination degree was the greatest, while in 2017, the impact was the least. This meant that with the rise of income level, tourist’s purchasing power and travel frequency increased accordingly, which ultimately led to a rapid increase in carbon emissions and the influence of economic development level on coupling coordination decreased. (2) The impact of urbanization level also tended to decrease. In 2010, it exerted the greatest influence on the coupling coordination degree of three tourism systems, and then gradually weakened. This was due to the coordinated advancement of the rural revitalization strategy and the new urbanization strategy, making rural areas with weak infrastructure and poor technology and economy become the main exchange places for tourism products. Consequently, the urbanization level had a reduced impact on the coupling coordination degree. (3) The impact of industrial structure declined. Except for failing the significance test in 2016, the industrial structure was significant in all other years which showed that the industrial structure was strongly related to coupling coordination degree. However, its impact was gradually decreasing and after reaching the minimum in 2016, whichhad a great deal to do with the adjustment of industrial structure of each province in China. (4) The impact of technological innovation level rose slowly. During the study period, its q value remained above 0.5576, which had the greatest impact on the coupling coordination degree. This indicated that technological innovation which was an important driving force for improving the coupling coordination degree was of great significance for completing infrastructure, improving energy utilization efficiency and promoting economic green development. (5) The impact of regional environmental pollution control level has increased. Its q value fluctuated between 0.2753 and 0.4966, with a relatively small influence. This showed that the increased investment in environmental pollution control was conducive to the promotion of clean energy and the innovation of low-carbon technology. Additionally, it had a positive effect on the low-carbon and healthy development of tourism and related industries, but it did not play a significant role.

Taken together, the driving factors that affected the coupling coordinated development of carbon emission, economic development and regional innovation of China’s tourism industry were as follows: technological innovation level, economic development level, urbanization level, regional environmental pollution control level, industrial structure. Among them, the technological innovation level and the regional environmental pollution control level had a gradually increasing trend. The economic development level and urbanization level showed a trend of first strengthening and then weakening, while the impact of industrial structure was weakened in the later period.

## 5. Conclusions

The coupling coordination relationship of carbon emission, economic development and regional innovation in tourism industry is of great significance to the sustainable development of tourism. Taking 30 provinces of China as an example, this paper established a comprehensive development index system of three major systems in the tourism industry. Through the coupling coordination degree model, dynamic degree and geographical detector, the spatiotemporal evolution and driving factors of coupling coordination development among the three were studied.

During the study period, the carbon emission efficiency of China’s tourism industry showed a reciprocating trend of “rising-falling”. The main reason was the increase or decrease of pure technical efficiency, which had little to do with the scale of production. As far as provinces were concerned, except for Tianjin, Jiangsu, Guangdong, and Guizhou, where the tourism carbon emission efficiency value was greater than 1, there was still a lot of room for improvement in tourism carbon emission reduction in other provinces.

From 2007 to 2017, the coupling coordination degree of three major systems in China’s tourism industry showed a good trend of steady rise. The major rank type in these provinces has undergone a transition from approaching imbalance to reluctant coordination to primary coordination that meant the coupling coordination degree between systems was developing in a good direction. The dynamic degree of coupling coordination development in each province was positive, the variation difference between provinces was small and the convergence characteristic was significant. There were obvious regional differences in the coupling coordination degree of three major systems in China, showing a gradient distribution pattern of “high in the east and low in the west”.

The carbon emission–economic development–regional innovation coupling coordination degree in tourism industry was affected by many aspects and its impact showed a certain difference in different study periods. In general, the significant factors that affected the coupling coordination degree of three major systems in China’s tourism industry were as follows: technological innovation level, economic development level, urbanization level, regional environmental pollution control level, and industrial structure.

Based on the above conclusions, we can find that the coupling coordination development of carbon emission–economic development–regional innovation in China’s tourism industry has turned towards a good direction as a whole, but there are still some problems. Therefore, the following suggestions are proposed:

(1) Clarify the direction of efforts to improve the tourism carbon emission efficiency. As the carbon emission efficiency of the tourism industry in most provinces has not reached the production frontier, there is large room for improvement in technological progress, energy conservation and emission reduction. For regions with high carbon emission efficiency of tourism, they should make full use of the radiation effect of high efficiency areas to realize the regional equalization of tourism resource allocation. Provinces with low technical efficiency should actively adjust the input–output ratio of resource elements, optimize emission reduction technology and improve element utilization ratio. Provinces with low scale efficiency should actively increase the amount of tourism input, improve the quality of tourism input and realize the rapid growth of tourism output.

(2) Narrow the development gap of coupling coordination between provinces. Provinces should formulate differentiated and precise tourism development policies in light of the actual conditions of all provinces and regions. By giving play to the comparative advantages of different regions, regional tourism cooperation and technical exchanges should be strengthened, and the rational flow of human resources and capital should be promoted. The eastern region should rely on its own economic strength to improve regional innovation level and accelerate the optimization and upgrading of industry. By actively absorbing the spillover from the east, the central and western regions complement the weak links of development and promote the good development of the coupling coordination degree of tourism three major systems which realize the sustainable and healthy growth of China’s overall tourism economy under environmental constraints.

(3) Strengthen the positive influence of driving factors on the improvement of coupling coordination. The improvement of coupling coordination degree of three major systems in tourism industry is a comprehensive project. While providing financial support, government departments should also strengthen technical support. On the one hand, the share of tourism investment in environmental pollution governance should be increased. By building green tourism infrastructure and superstructure, and establishing an environmental management system of tourism, the ecological governance of tourism branches such as tourism traffic, tourism accommodation will be strengthened. On the other hand, government departments should increase investment in low-carbon technologies, and actively encourage the development and introduction of low-carbon technologies. To promote the upgrading of energy consumption structure and the transformation of economic development, the tourism industry will achieve a comprehensive and efficient sustainable development.

However, this study has certain limitations. First, due to data limitations, this paper only considered the provincial level in the research scope which reduced the regional differences to some extent. Therefore, taking the prefecture-level cities and counties as the research objects, it not only makes the results more convincing, but has practical significance for targeted formulation of regional emission reduction policies and effective implementation of emission reduction targets. Second, the selection of system evaluation indexes was not comprehensive. The development level of three major systems in tourism industry is affected by many aspects, but the index system was constructed in this paper from only a few aspects lacking certain rationality that needs to be further improved in future research. Third, the coupling coordination degree of a region is not only affected by economy and technology, but also affected by neighboring provinces. Therefore, the discussion on spatial correlation and heterogeneity of coupling coordination degree of three major systems in tourism industry is also one of the emphases of future research.

## Figures and Tables

**Table 1:** Reference coefficient of various energy discount standard coal.

Energy Type	Convert Coefficient	Energy Type	Convert Coefficient
Raw coal	0.7143 kgce/kg	Liquefied petroleum gas	1.7143 kgce/kg
Briquette	0.500–0.700 kgce/kg	Oilfield natural gas	1.3300 kgce/m^3^
Coke	0.9714 kgce/kg	Gasfield natural gas	1.2143 kgce/m^3^
Crude	1.4286 kgce/kg	Liquified natural gas	1.7572 kgce/kg
Fuel oil	1.4286 kgce/kg	Coke oven gas	0.5714–0.6143 kgce/m^3^
Gasoline	1.4714 kgce/kg	Blast furnace gas	0.1286 kgce/m^3^
Kerosene	1.4714 kgce/kg	Heat (equivalent value)	0.03412 kgce/MJ
Diesel oil	1.4571 kgce/kg	Electricity (equivalent value)	0.1229 kgce/(kw·h)

Note: In this paper, the reference coefficient of natural gas discount standard coal is taken as the average value of oil field natural gas and gas field natural gas of 1.2722 kgce/m^3^. Besides, the reference coefficient of briquette and coke oven gas discount standard coal are also averaged as 0.6000 and 0.59285 kgce/kg, respectively.

**Table 2:** The comprehensive evaluation indexes of three subsystems.

Subsystem	First-Class Index	Second-Class Index	Weight
Tourism carbon emission	Tourism carbon emission efficiency	Comprehensive efficiency of tourism carbon emission	0.1737
		Pure technical efficiency of tourism carbon emission	0.1252
		Scale efficiency of tourism carbon emission	0.1640
	Current situation of tourism carbon emission	Tourism carbon emission intensity (t/10,000 CNY)	0.1144
		Tourism carbon emission productivity (CNY/t)	0.1320
		Tourism carbon emission density (t/km^2^)	0.1517
		Per capita tourism carbon emissions (t/person)	0.1390
Tourism economic	Tourism economic scale	Total tourism revenue (100 million yuan)	0.1494
		Number of tourists (10,000 people)	0.1293
		Tourism income per capita (CNY)	0.1323
	Tourism economic structure	Proportion of tourism revenue in GDP (%)	0.0786
		Proportion of tourism foreign exchange income in total tourism revenue (%)	0.1319
		Proportion of inbound tourists in total tourists (%)	0.1730
	Tourism economic vitality	Total tourism revenue growth rate (%)	0.0180
		Number of tourism employees (person)	0.1087
		Tourism consumption expenditure per capita (CNY/person)	0.0788
Tourism innovation	Tourism innovation input	Proportion of tourism R&D expenditure in GDP (%)	0.1682
		Investment in tourism fixed assets per capita (CNY/person)	0.1245
		Number of tourism colleges and universities (unit)	0.0897
	Tourism innovation output	Technology market technology contract amount (10,000 CNY)	0.1169
		Granted amount of tourism invention patents (number)	0.0854
		Tourism total labor productivity (10,000 CNY/person)	0.1102
	Tourism innovation benefits	Tourism enterprises revenue (10,000 CNY)	0.1612
		Proportion of total tourism revenue in the added value of tertiary industry (%)	0.0620
		Tourism energy consumption intensity (t/10,000 CNY)	0.0819

**Table 3:** The division standard for coupling coordination degree.

Imbalanced Recession		Coordinated Development	
Scoring Standard	Classification	Scoring Standard	Classification
0.00~0.09	Extreme imbalance	0.50~0.59	Reluctant coordination
0.10~0.19	Severe imbalance	0.60~0.69	Primary coordination
0.20~0.29	Moderate imbalance	0.70~0.79	Intermediate coordination
0.30~0.39	Slight imbalance	0.80~0.89	Well coordination
0.40~0.49	Approaching imbalance	0.90~1.00	High coordination

**Table 4:** China’s provincial tourism carbon emission efficiency calculation results.

Province	Comprehensive Efficiency	Pure Technical Efficiency	Scale Efficiency
Beijing	0.5810	0.5850	0.9752
Tianjin	1.1172	1.1588	0.9647
Hebei	0.4637	0.4864	0.9541
Shanxi	0.5235	0.5513	0.9525
Inner Mongolia	0.6384	0.7078	0.9196
Liaoning	0.4477	0.5480	0.8994
Jilin	0.5618	0.5920	0.9403
Heilongjiang	0.5798	0.6388	0.9070
Shanghai	0.7073	0.7446	0.9519
Jiangsu	1.2743	1.4224	0.9068
Zhejiang	0.6219	0.6317	0.9835
Anhui	0.7140	0.7804	0.9155
Fujian	0.8825	0.9527	0.9229
Jiangxi	0.7501	0.8386	0.8924
Shandong	0.5484	0.5516	0.9944
Henan	0.9159	0.9646	0.9461
Hubei	0.7184	0.7547	0.9535
Hunan	0.6874	0.7171	0.9587
Guandong	1.0841	1.1879	0.9123
Guangxi	0.7180	0.7955	0.9050
Hainan	0.6111	1.0489	0.6025
Chongqing	0.6811	0.7919	0.8659
Sichuan	0.7824	0.8104	0.9649
Guizhou	1.0492	1.1063	0.9512
Yunnan	0.6086	0.6670	0.9175
Shaanxi	0.5690	0.6101	0.9328
Gansu	0.4653	0.7614	0.6542
Qinghai	0.4352	1.5010	0.300
Ningxia	0.2944	0.8811	0.3982
Xinjiang	0.4516	0.5469	0.8275

**Table 5:** Detection results of factors affecting the coupling coordination degree.

Year	X1	X2	X3	X4	X5
2007	0.4852	0.3863	0.2831	0.5576	0.2753
2008	0.4624	0.4334	0.3434	0.6688	0.4566
2009	0.5597	0.5342	0.2499	0.6857	0.4851
2010	0.5050	0.5555	0.2970	0.7117	0.4588
2011	0.5514	0.4283	0.3054	0.7362	0.3763
2012	0.3889	0.5186	0.2793	0.7375	0.2996
2013	0.4805	0.4983	0.2376	0.6918	0.4630
2014	0.4390	0.5348	0.2041	0.7211	0.4966
2015	0.3395	0.3657	0.0844	0.7444	0.3127
2016	0.3314	0.2880	0.0326 *	0.6911	0.2778
2017	0.2548	0.2469	0.0913	0.6036	0.3709

Note: * means that it has not passed the significance test and not marked * means it has passed the 1% significance test.
